# The Alleviating Effect of Abalone Viscera Collagen Peptide in DSS-Induced Colitis Mice: Effect on Inflammatory Cytokines, Oxidative Stress, and Gut Microbiota

**DOI:** 10.3390/nu17111926

**Published:** 2025-06-04

**Authors:** Binxiong Liu, Lili Liu, Chunjiang Li, Tengming Guo, Changcheng Li, Meiling Tian, Ting Fang

**Affiliations:** 1 College of Food Science, Fujian Agriculture and Forestry University, Fuzhou 350002, China; lbx_zhou@163.com (B.L.); 15160583408@163.com (L.L.); lichunjiang323@163.com (C.L.); 13107657387@163.com (T.G.); changcheng_li@fafu.edu.cn (C.L.); 2 National R&D Center for Vegetable Processing, Fuzhou 350002, China

**Keywords:** abalone viscera, collagen peptide, gut microbiota, ulcerative colitis, inflammatory cytokine, oxidative stress

## Abstract

Background/Objectives: Abalone viscera is a discarded seafood by-product that contains a wealth of protein and is a good source of collagen peptides which have proven to have great potential in ameliorating host inflammation. The present study was conducted to evaluate the anti-inflammatory capacity of collagen peptide extracted from abalone viscera. Methods: Low, medium, and high dosages (300, 600, and 900 mg/kg/d) of abalone viscera collagen peptide (AVCP) were orally administered to DSS-induced acute colitis mice. The inflammatory mediators and oxidative stress factors were assessed using the ELISA method, and gut microbiota was widely studied by 16S rRNA sequencing technology. Results: The results showed that oral administration of AVCP led to a significant alleviation of weight loss, colon length shortening, and DAI escalation in colitis mice. AVCP could also alleviate the pathological damage of colon tissue; inhibit splenic edema and thymic atrophy; reduce the serum level of inflammatory mediators (IL-1β, IL-6, TNF-α, IL-17A, and myeloperoxidase (MPO)); and improved antioxidant capacity (the activity of superoxide dismutase (SOD) and glutathione peroxidase (GSH-Px) increased and malondialdehyde (MDA) level decreased). Moreover, AVCP restored the balance of the gut microbiota, such as *Escherichia-Shigella*, *Bacteroides*, *norank_f_Muribaculaceae*, *Rikenellaceae_RC9_gut_group*, and *Parasutterella*. Conclusions: Collectively, our observations elucidated the potential use of AVCP as a prebiotic for ulcerative colitis alleviation.

## 1. Introduction

Ulcerative colitis (UC) is one of the two primary forms of inflammatory bowel disease (IBD) that mainly affects the colon and rectum [1]. This disease is most common in Western industrialized countries, and its occurrence and frequency are increasing worldwide, particularly in newly industrialized nations such as China, India, and Latin America [2,3]. The worldwide prevalence of UC was around 5 million in 2023 [3]. Symptoms of UC in 90% of patients are rectal bleeding or bloody diarrhea [3]. Other symptoms are fatigue, abdominal pain, and bowel urgency, which greatly reduce the quality of life for patients [4]. The repeated and long-term chronic inflammation in UC leads to a higher risk of colorectal cancer [5]. Although the etiology of UC is not yet fully articulated, current research has emphasized the importance of the immune-inflammatory response, intestinal mucus barrier, and intestinal flora [6,7]. Moreover, there is no cure for UC, and current treatment drugs such as corticosteroids, antibiotics, and immunosuppressants can lead to long-lasting negative side effects [1]. Consequently, there is an urgent need to find safe and effective strategies to prevent and mitigate the onset and progression of UC.

Abalone viscera is a discarded by-product of seafood with high nutritional value that contains abundant polysaccharides, proteins, taurine, and trace elements [8,9]. Studies have demonstrated that the extracts and enzymatic hydrolysates obtained from abalone viscera have a variety of physiological benefits, including antioxidant, immunomodulatory, and cytoprotective activities [8,9]. China is the largest producer and consumer of farmed abalone (*genus Abalone*, *family Abaloneidae*, *order Archaeopteryx*). In 2023, the annual abalone production of China reaches to 24.50 × 10^4^ tons [10]. However, approximately 90% of the current deep processing of abalone focuses on its gastropods, resulting in the wasteful of the abalone viscera, which accounts for 15–25% of the total abalone weight to be wasted [8,11]. This resulted not only in a waste of valuable resources but also in environmental pollution. Thus, it is urgent to fully and reasonably utilize the abalone viscera resources. Recent investigations have revealed that the proteins rich in abalone viscera have been used as precursors of bioactive peptides that displayed significant antioxidant, antitumor, antimicrobial, and angiotensin-I converting enzyme (ACE) inhibitory capacity [12,13,14,15]. Hu et al. prepared abalone (*Haliotis discus hannai*) visceral peptides using proteases showed excellent in vitro antioxidant activity, and the antioxidant capacity of the fraction with peptides < 1 kDa were significantly higher than fraction containing peptides > 1 kDa [14]. Moreover, peptides possessing both Tyr and Cys had the strongest in vitro and LO2 cellular antioxidation compared to other peptides [12]. Rivera-Pérez et al. demonstrated that abalone (*Haliotis fulgens*) visceral peptide with a molecular weight size of 61.2–31 kDa can inhibit human prostate cancer cells by 50% and a molecular weight size of 66.2–116.25 kDa exhibited antimicrobial activity against *Pseudomona aeuroginosa* [13]. Heo et al. found that abalone visceral peptides prepared with trypsin displayed a prominent ACE inhibitory activity [15]. The fermented blacklip abalone (*Haliotis ruber*) viscera using *Aspergillus oryzae* 001 had a stronger inhibitory effect on ACE activity and on the elevation of blood pressure in spontaneously hypertensive rats [16]. These suggest that abalone viscera peptides may be promising ingredients for functional foods.

Abalone contains a large amount of collagen, which is normally situated in the extracellular matrix of connective tissues and offers an insoluble support for the shape and morphology of abalone muscle [17]. In abalone viscera, collagen is one of the most abundant proteins in its connective tissue [18]. Therefore, abalone visceral connective tissue provides potential high-quality raw material for the manufacture of collagen peptides. Collagen can be enzymatically hydrolyzed to release biologically active peptides (collagen peptides) with various physiological functions, including antioxidant, immunomodulatory, antiaging, and wound-healing activities [19,20]. Collagen peptides have been broadly investigated in vivo and clinically for their pronounced protective effects on the skin [19,21]. Recently, research has demonstrated that collagen peptides from cod (*Gadus*) skin significantly attenuated the inflammatory response, recovered mucosal barrier function, and suppressed necrosis in dextran sulfate sodium (DSS)-induced mice colitis [22]. Cod skin collagen peptides may exert anti-inflammatory effects by inhibiting the NF-κB/MAPK signaling pathway [23]. However, few reports have investigated the preparation of functional peptides using abalone visceral connective tissue, nor have the active peptides been defined as collagen peptides. The in-depth exploration of this resource is urgently needed by evaluating its collagen peptide bioactivity.

To address the problem of massive discard of abalone viscera, we enzymatically extracted collagen peptides from abalone viscera connective tissue. Moreover, given the already well-characterized in vitro and in vivo antioxidant properties of abalone viscera peptides, it is hypothesized that abalone viscera collagen peptides could exhibit anti-inflammatory against colitis. The anti-inflammatory activity of abalone viscera collagen peptide (AVCP) in mitigating ulcerative colitis was conducted in a mouse colitis model established by DSS. This study contributes to the promotion of the development and utilization of abalone viscera and provides a scientific basis for the development of functional foods based on collagen peptides.

## 2. Materials and Methods

### 2.1. AVCP Preparation

Abalone viscera were kindly supplied by Zhao’an Hailian Food Co., Ltd. (Zhangzhou, China). The abalone viscera were pulped using a single-cylinder pulper (DJ1-0.12, Jiangsu Kewei Machinery Co., Ltd., Jiangsu, China), which could crush the abalone offal and leave the tough connective tissue. The connective tissue was then immersed in a 0.1 mol/L NaOH solution for 15 min, washed until pH = 9, and homogenized using colloid mills (JMS80, Wenzhou Mutual Feng Machinery Co., Ltd., Wenzhou, Zhejiang, China). The connective tissue slurry was treated with Alkaline Protease of 6000 U/g (Solarbio Co., Ltd., Beijing, China) and incubated in a water bath maintained at 55 °C for a period of 4 h under conditions of constant agitation (Magnetic Stirrer DF-101S, Shanghai Lichen Bangxi Instrument Technology Co., Ltd., Shanghai, China). Then, the hydrolysate was boiled at 90 °C for 10 min to inactivate the enzyme, cooled to room temperature, and centrifuged at 4000× *g* for 15 min. The resulting supernatant was collected and lyophilized for further analysis.

### 2.2. Molecular Weight Distribution Analysis

The molecular weight distribution (MWD) of AVCP was performed on the implementation of gel permeation chromatography (GPC) on an Agilent 1260 Infinity II liquid chromatography system (Agilent Technologies, SC, CA, USA) outfitted with a TSK-GEL G2000 SWXL gel chromatographic column (300 mm × 7.8 mm, 5 μm) (TOSOH Bioscience, Tokyo, Japan). AVCP power was dissolved in the PBS (3 mg/mL), filtered by a 0.45 µm filter, and then analyzed using an isocratic elution with 0.5% Trifluoro-acetic acid (TFA) in 40% acetonitrile at a flow rate of 1.0 mL/min. The injection volume was 10 µL, the column temperature was 40 °C, and the detection wavelength was 220 nm. The column was standardized with the following standard proteins (Sigma-Aldrich, St. Louis, MO, USA): glycine-glycine-tyrosine-arginine (451 Da), glycine-glycine-glycine (189 Da), bacitracin (1423 Da), aprotinin (6512 Da), and cytochrome C (12,384 Da).

### 2.3. Hydroxyproline Content Analysis

The hydroxyproline content of AVCP was quantified using a hydroxyproline assay kit (BC0250, Solarbio Co., Ltd., Beijing, China) according to the manufacturer’s instructions.

### 2.4. Animal Experiments

The 54 male C57BL/6J mice (20 ± 1 g) were obtained from Beijing HFK Bio-Technology. Co., Ltd. (Beijing, China) (Animal Certificate Number: SCXK(Jing) 2024-003) and housed under a specific pathogen-free (SPF) animal facility (12/12-h light/dark cycle, relative humidity 60 ± 5%, and temperature 24 ± 1 °C) with free access to clean water and a standard diet. All animal experiments were executed according to the guidelines that had been approved by the Ethics Committee of Experimental Animal Care at Fujian Agriculture and Forestry University (Approval number: PZCASFAFU24098. Date: 15 March 2024).

**Figure 1:**
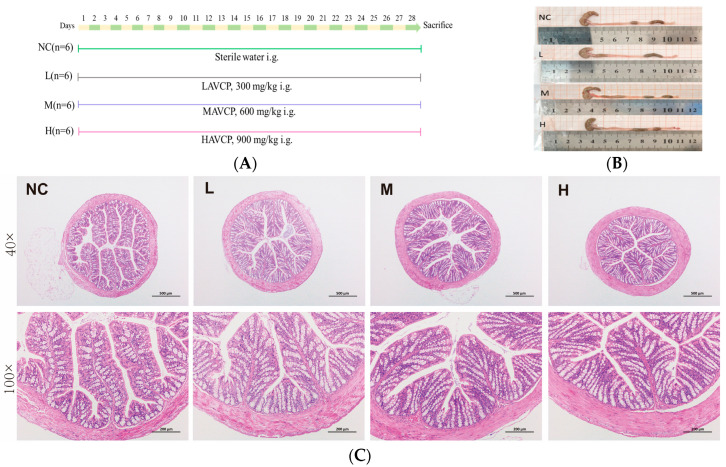

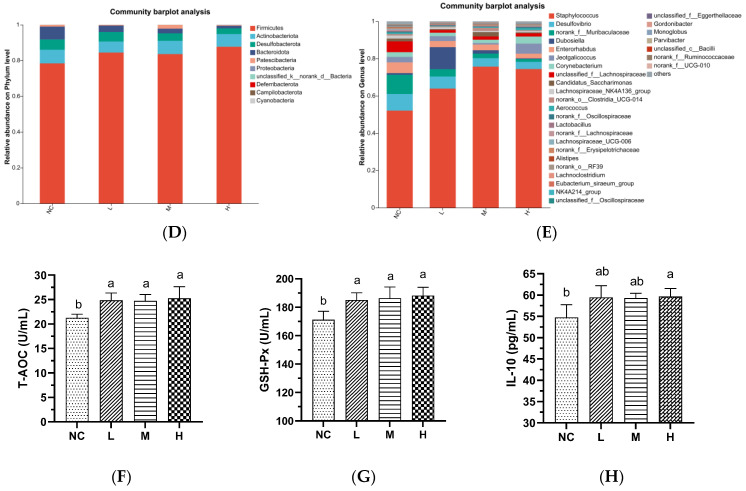
(**A**) Flowchart of the animal experiment design (*n* = 6/group). (**B**) Typical picture of intestinal appearance. (**C**) H&E staining of representative histology sections (40×, Scale bar 500 μm; 100×, scale bar 200 μm). Relative abundance of dominant bacteria at the phylum (**D**) and genus (**E**) levels. The serum levels of (**F**) T-AOC, (**G**) GSH-Px, and (**H**) IL-10 in mice. Different lowercase letters indicate significant differences between groups (*p* < 0.05).

**Figure 2:**
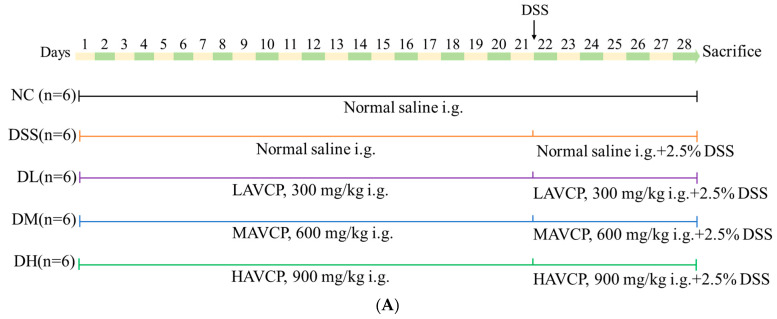

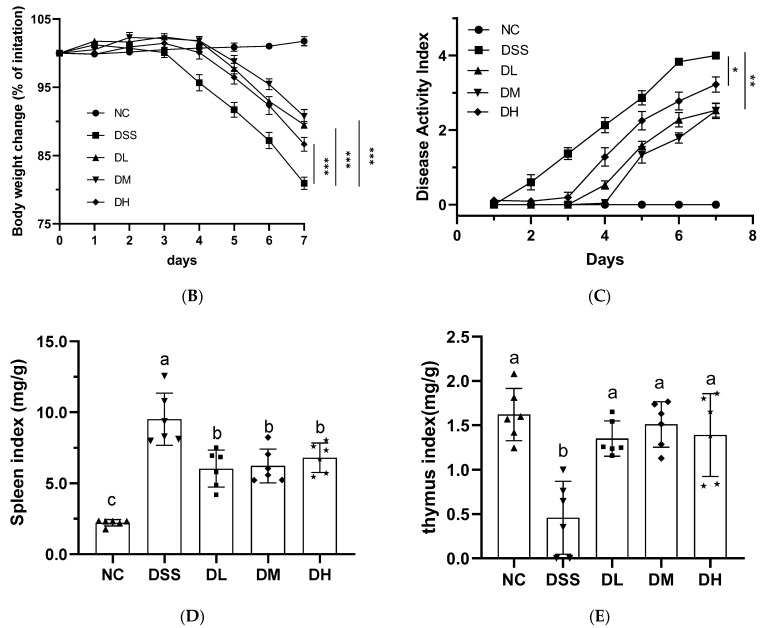
AVCP alleviates symptoms of DSS-induced colitis in mice. (**A**) Flowchart of the animal experiment design (*n* = 6/group). (**B**) Changes in body weight. (**C**) DAI scores. (**D**) Spleen index. (**E**) Thymus index. * *p* < 0.05, ** *p* < 0.01, and *** *p* < 0.001. Different lowercase letters indicate significant differences between groups (*p* < 0.05).

The flowchart of the animal experiment design is shown in Figure 1A and Figure 2A. Briefly, after a week of adaptation and rearing, mice were randomly divided into 8 experimental groups: normal control (NC), low-dose (L), medium-dose (M), and high-dose (H) AVCP treatment groups, colitis model (DSS), low-dose AVCP + DSS group (DL), medium-dose AVCP + DSS group (DM), and high-dose AVCP + DSS group (DH) (*n* = 12 for the NC group and *n* = 6 for the other group). The L and DL groups, M and DM groups, as well as H and DH groups, were orally administered with a low dose (300 mg/kg/day), medium dose (600 mg/kg/day), and high dose (900 mg/kg/day) of AVCP, respectively, for the whole experiments (days 1–28), while the control (NC) group and DSS groups were gavaged with the same volume of saline. For the last 7 days (days 22–28), the NC, L, M, and H groups were given clean water, and the colitis disease group (DSS, DL, DM, and DH) received 2.5% DSS (36,000–50,000 Da; MP Biomedicals, Solon, OH, USA) in water [24]. During the experiment, the mouse state, body weight, fecal traits, and fecal occult blood were assessed daily. The disease activity index (DAI) summarized the weight changes, fecal traits, and stool blood of mice with a scale that ranged from 0, indicating absence of disease symptoms, to 4, signifying severe disease manifestations according to the previous description [25]. After collecting blood from the orbital plexus, all mice were euthanized by cervical spine dislocation on the eighth day. The blood sample was then subjected to incubation at room temperature and centrifugation (1000× *g*, 10 min) to obtain the serum. About 1-cm colonic segments from the proximal colon were fixed with 4% paraformaldehyde and used for histological analysis. The spleen and thymus were collected for immune organ index analysis. The cecal contents were collected and immediately frozen in liquid nitrogen for follow-up 16S rRNA sequencing analysis.

### 2.5. Immune Organ Index Analysis

The target organs (spleen and thymus) were excised and immediately weighed in a controlled setting to ascertain the immune organ index. The formula is shown below:

$$\mathrm{Immune}\;\mathrm{organ}\;\mathrm{index}=\frac{\mathrm{organs}\;\mathrm{weight}\;(\mathrm{mg})}{\mathrm{body}\;\mathrm{weight}\;(g)}$$

### 2.6. Histopathological Analysis

The fixed colon tissues were processed through the embedding procedure in paraffin, then sectioned carefully, and finally stained with hematoxylin and eosin (H&E) (Servicebio Technology Co., Ltd., Wuhan, Hubei, China). Images were captured by an ECLIPSE E100 microscope (Nikon, Tokyo, Japan). The H&E-stained sections were subjected to blind scoring for the purpose of histological assessment on a scale of 1–12, as previously described [24].

### 2.7. Inflammatory Mediators and Oxidative Stress Factors Analysis

The contents of the inflammatory mediators (IL-1β, IL-6, TNF-α, IL-17A, IL-10, and myeloperoxidase (MPO)) and oxidative stress factors (malondialdehyde (MDA), total antioxidant capacity (T-AOC), superoxide dismutase (SOD), and glutathione peroxidase (GSH-Px)) in mice serum were conducted according to the instructions of the ELISA kit (Sino Best Biological Technology Co., Ltd., Shanghai, China).

### 2.8. 16S rRNA Sequencing of Gut Microbiota

16S rRNA gene sequence analyses were carried out by Majorbio Bio-Pharm Technology Co., Ltd. (Shanghai, China), as described previously [24]. Briefly, the genomic DNA from cecal contents were extracted and used as a template for the hypervariable region V3–V4 of the bacterial 16S rRNA gene with 338F (5′-ACTCCTACGGGAGGCAGCAG-3′) and 806R (5′-GGACTACHVGGGTWTCTAAT-3′) primer pairs. The amplified DNA pyrosequencing was performed on an Illumina Nextseq2000 platform. Finally, the Amplicon Sequence Variants (ASVs) that reflect the diversity of the gut microbiota composition in each sample were obtained. All data statistics, including α-diversity analysis, principal coordinate analysis (PCoA), non-metric multidimensional scaling (NMDS), correlation analyses, and differential bacterial abundance analysis were performed on the free online Majorbio Cloud Platform (https://cloud.majorbio.com/page/project/overview.html, accessed on 1 March 2025). The raw sequence data in this study have been deposited in the NCBI Short Read Archive database under the BioProject accession number PRJNA1236735 (data will be made public on 28 February 2029).

### 2.9. Statistical Analysis

Data are expressed as the mean ± SEM. The difference between groups was processed with the SPSS 20.0 software system using a one-way analysis of variance (ANOVA) followed by Tukey’s multiple comparison test. The difference between groups was significant at *p* < 0.05. Unless otherwise stated, the figures were plotted using GraphPad Prism software (7.0 version, GraphPad Software, La Jolla, CA, USA).

## 3. Results

### 3.1. Characterization and In Vivo Safety Evaluation of AVCP

The molecular weight distribution of AVCP is presented in Table 1. As can be seen from the table, the molecular weight ranges are <1000, 1000–2000, 2000–3000, 3000–5000, 5000–10,000, and >10,000, which account for 19.3%, 21.0%, 25.6%, 23.1%, 8.1%, and 2.9%, respectively. The <10,000 Da group accounted for 97.1%, which is higher than the national standard GB31645-2018 on collagen peptides, which states those peptides smaller than 10,000 Da account for ≥90% [26]. Moreover, the hydroxyproline content of AVCP was 6.56 ± 0.23%, which was higher than the hydroxyproline content in collagen peptides of >3.0%, as stipulated in the national standard GB31645-2018 [26]. These results suggest that the AVCP obtained in this study is a high-quality collagen peptide.

We further evaluated the in vivo safety of AVCP, and the results showed that different doses of AVCP (300, 600, and 900 mg/kg/d) had no significant effects on body weight changes; organ (heart, liver, kidney, spleen, and thymus) indices; colon length and histopathology; serum levels of IL-1β, IL-6, TNF-α, MDA and SOD activity; and relative abundance of gut microbiota at the phylum and genus levels of healthy mice (Figure 1A–E and Figures S1 and S2; Tables S1 and S2). However, AVCP administration significantly increase the T-AOC, GSH-Px activity, and IL-10 level in mice serum (Figure 1F–H). These suggest that AVCP is non-toxic and has certain antioxidant and anti-inflammatory activities in healthy mice, being a good source of functional food.

### 3.2. AVCP Administration Alleviates DSS-Induced Acute Colitis

#### 3.2.1. Influence of AVCP on Body Weight, DAI, and Organ Index

Clinical manifestations of weight loss, diarrhea, and bloody stools in mice induced by DSS are similar to those of UC [27]. The enlarged spleens and thymic atrophy in mice caused by inflammation and immunosuppression induced by DSS are also typical indicators of UC [25]. In this study, the alleviating effects of AVCP against colitis were investigated in a DSS-induced acute colitis mouse model (Figure 2A). DSS administration significantly decreased the body weight (from the fourth day) and thymus index and increased the DAI score (from the third day) and spleen index of mice compared to the control group (Figure 2B–E). Nevertheless, orally supplemented with different doses of AVCP (300, 600, and 900 mg/kg/d) significantly reduced DSS-induced weight loss, diarrhea, bloody stools, splenomegaly, and thymic atrophy (Figure 2B–E). Moreover, the three treatment groups showed no significant differences in resistance to splenic edema and thymus suppression (Figure 2B–E). These findings indicate that AVCP can inhibit DSS-induced acute colitis in mice.

#### 3.2.2. Influence of AVCP on Colon Histopathology

**Figure 3:**
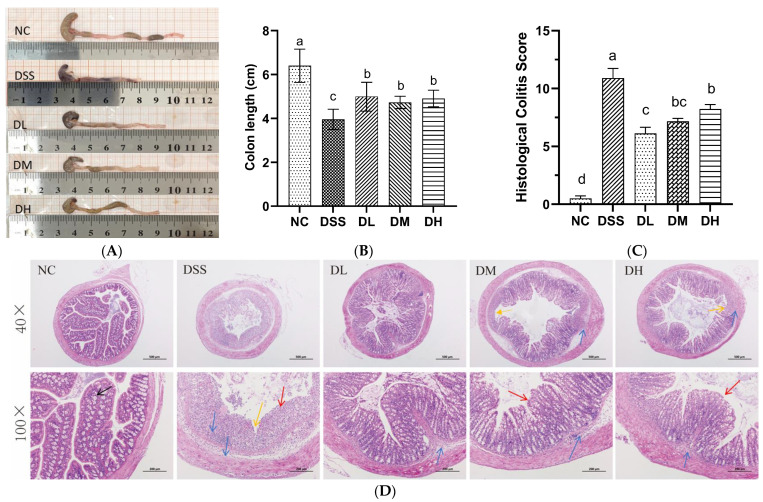
(**A**) Typical picture of intestinal appearance. (**B**) Colon length. (**C**) Histopathological colitis score. (**D**) H&E staining of representative histology sections (40×, Scale bar 500 μm; 100×, scale bar 200 μm). Note the presence of goblet cells (black arrows), inflammatory cell infiltrates (blue arrows), crypt damage (yellow arrows), and goblet cell loss (red arrows). Significant differences between groups are indicated by different lowercase letters (*p* < 0.05).

The colonic pathological variations during UC progression include colonic shortening, goblet cell loss, crypt destruction, and neutrophil infiltration at the lesion site [24]. As expected, the colon of DSS-induced colitis mice showed significant colonic shortening, intestinal mucosal hemorrhage, goblet cell and crypt destruction, and extensive neutrophil infiltration (Figure 3A–D). In contrast, colitis mice treated with different doses of AVCP (300, 600, and 900 mg/kg/day) significantly counteracted colonic shortening; presented relatively intact colonic crypts, goblet cell, and intestinal epithelium; and decreased inflammatory infiltration (Figure 3A,D). Precisely, DL, DM, and DH treatment groups significantly reduced colon shortening caused by DSS by 27%, 24%, and 20%, respectively (Figure 3B), but no significant difference was observed among the three treatment groups. Meanwhile, the DL treatment showed the most significant reduction in the histopathological scores and less inflammatory cell infiltration in the colon tissues of the DL group than in the other groups (Figure 3C,D). These findings indicate that AVCP attenuates DSS-induced acute colitis in mice.

#### 3.2.3. Influence of AVCP on Inflammatory Mediators in Serum

**Figure 4:**
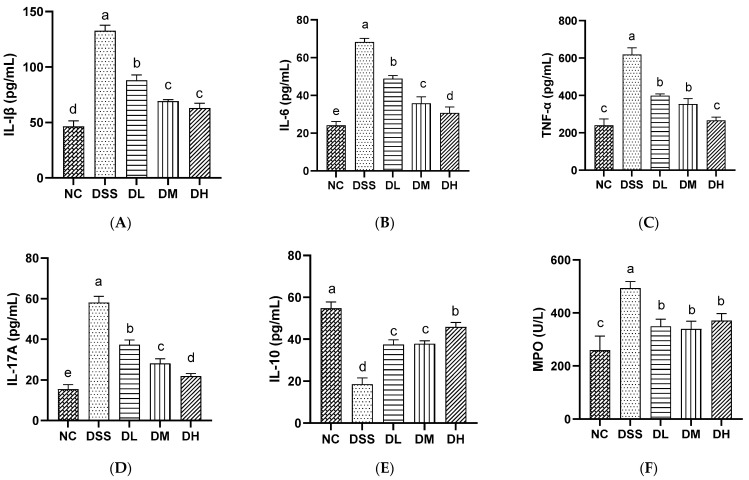
The serum levels of (**A**) IL-1β, (**B**) IL-6, (**C**) TNF-α, (**D**) IL-17A, (**E**) IL-10, and (**F**) MPO in mice. Different lowercase letters indicate significant differences between groups (*p* < 0.05).

The imbalance between proinflammatory and anti-inflammatory cytokines is an important characterization for the development of UC [28]. MPO is a marker to verify neutrophil recruitment, where high neutrophils are a sign of an inflammatory response [29]. In our study, the serum levels of proinflammatory cytokines IL-1β, IL-6, TNF-α, and IL-17A were significantly up-regulated and the anti-inflammatory cytokines IL-10 were significantly down-regulated in mice treated with 2.5% DSS water (Figure 4A–E). DSS also significantly increased the serum MPO levels compared to normal mice (Figure 4F). The intervention of mice with three doses of AVCP (300, 600, and 900 mg/kg/day) significantly inhibited the elevation of the IL-1β, IL-6, TNF-α, IL-17A, and MPO levels and the reduction of the IL-10 level (Figure 4A–F). Notably, a high dose of AVCP had more pronounced inhibitory effects on IL-6, TNF-α, and IL-17A and a more significant promotional effect on IL-10 than the other dose groups. The effects of AVCP appear to be dose-dependent. Nevertheless, there was no significant difference among the three doses of AVCP on the MPO levels (Figure 4B–F). The results demonstrated that AVCP exhibited an anti-inflammatory effect, as evidenced by its inhibition of the IL-1β, IL-6, TNF-α, and IL-17A levels while concomitantly promoting the IL-10 level.

#### 3.2.4. Influence of AVCP on Oxidative Stress Factors in Serum

**Figure 5:**
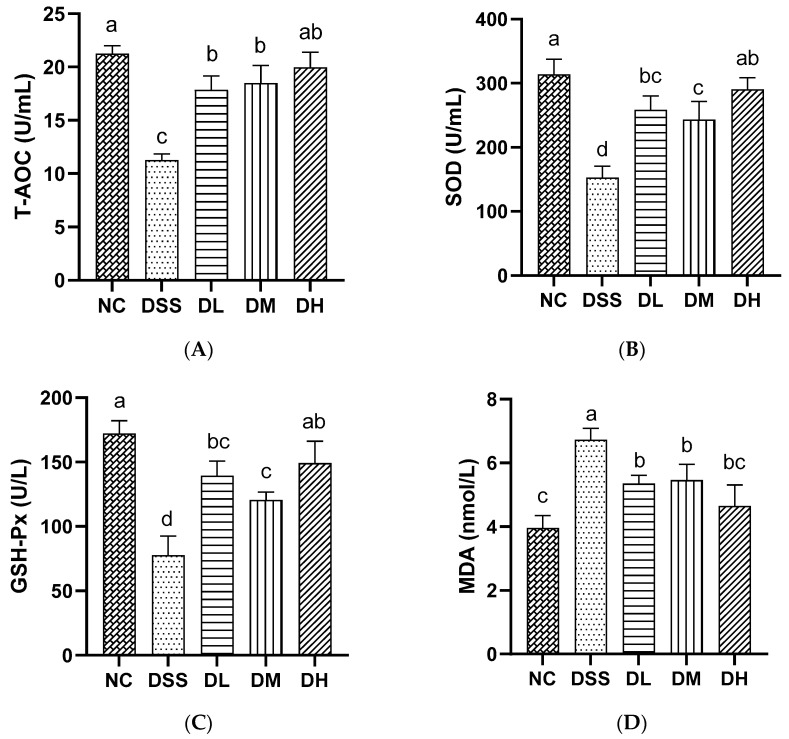
The serum levels of (**A**) T-AOC, (**B**) SOD, (**C**) GSH-Px, and (**D**) MDA in mice. Different lowercase letters indicate significant differences between groups (*p* < 0.05).

Antioxidant enzymes reduce intracellular oxidative stress by using their specific substrates to reduce oxidants, which then reduce inflammatory responses [30]. MDA is an oxidative stress biomarker that can be measured in the blood and feces of patients with IBD, and its level correlates with the severity of the disease [31]. In this study, the serum level of MDA was markedly elevated in the DSS group, while the activity of T-AOC, SOD, and GSH-Px was significantly reduced (Figure 5A–D). The oral administration of AVCP (300, 600, and 900 mg/kg/day) significantly reduced the MDA levels and increasing T-AOC, SOD, and GSH-Px activities, thereby significantly attenuating oxidative stress in colitis mice (Figure 5A–D). The results showed that AVCP could improve the antioxidant capacity, thereby exerting an anti-inflammatory effect and alleviating intestinal inflammation.

### 3.3. AVCP Administration Alleviates DSS-Induced Gut Microbiota Dysbiosis

#### 3.3.1. Alpha and Beta-Diversity Analysis of Gut Microbiota

**Figure 6:**
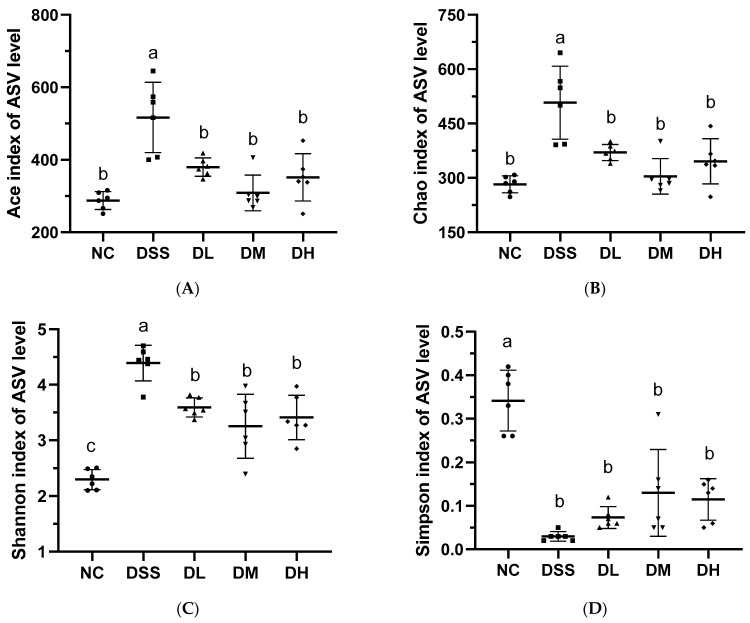
Alpha diversity of gut microbiota. (**A**) Ace Index. (**B**) Chao Index. (**C**) Shannon Index. (**D**) Simpson Index. Different lowercase letters indicate significant differences between groups (*p* < 0.05).

**Figure 7:**
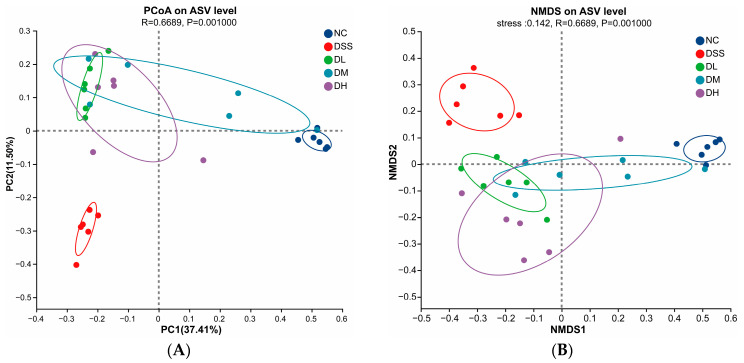
Beta diversity analysis of gut microbiota. (**A**) PCoA analysis. (**B**) NMDS analysis.

Major changes in the membership and function of gut microbiota that promote potential disease states (dysbiosis) are prevalent in patients with UC [32]. In this study, 16S rRNA sequencing was used to examine how AVCP affects the diversity and composition of gut microbes in colitis mice. For alpha diversity, the Chao, Ace, and Shannon Indices were significantly elevated, and the Simpson Index was significantly reduced after DSS administration (Figure 6), indicating a significant increase of alpha diversity. However, AVCP (300, 600, and 900 mg/kg/day) treatment considerably decreased the Chao, Ace, and Shannon Indices and increased the Simpson Index compared to the DSS group (Figure 6), inferring that AVCP could restore the alpha diversity changed caused by DSS administration. Both principal coordinates analysis (PCoA) and non-metric multidimensional scaling (NMDS) data that characterize the beta diversity of the gut microbiota showed unique gut microbiota structures in the NC and DSS groups and AVCP-treated groups far removed from the DSS group while closed to the NC group (Figure 7). These infer that AVCP could significantly alleviate DSS-induced gut microbiota composition changes in colitis mice.

#### 3.3.2. Composition of Gut Microbiota

**Figure 8:**
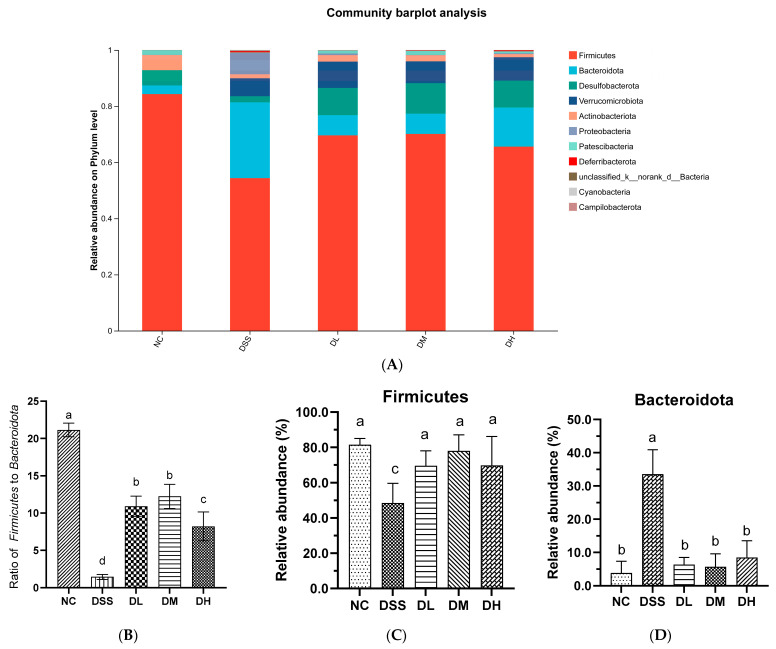

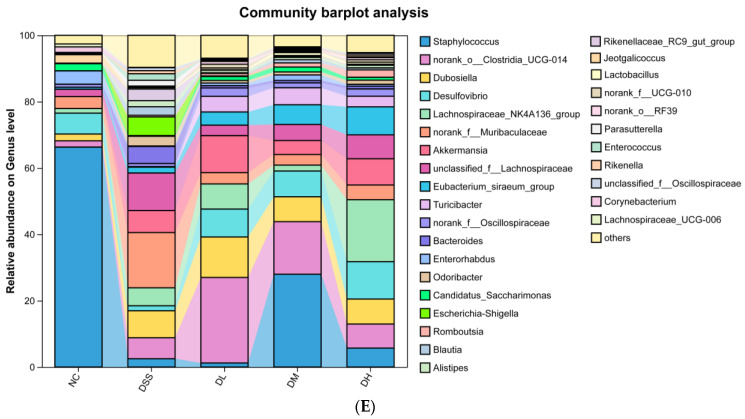
AVCP regulates gut microbiota in colitis mice. (**A**) Relative abundance of dominant bacteria at the phylum level. (**B**) Ratio of Firmicutes to Bacteroidota. (**C**) Relative abundance of Firmicutes. (**D**) Relative abundance of Bacteroidota. (**E**) Relative abundance of dominant bacteria at the genus level. Significant differences between groups are denoted by different lowercase letters (*p* < 0.5).

The gut microbiota composition across all groups was further analyzed, and the results were presented in Figure 8. At the phylum level, six dominant bacterial phyla, including Firmicutes, Bacteroidota, Desulfobacterota, Verrucomicrobiota, Actinobacteriota, and Proteobacteria, were identified (Figure 8A). The ratio of Firmicutes to Bacteroides decreased significantly compared to the NC group (Figure 8B), which is in agreement with prior research demonstrating a significantly reduced F/B ratio in individuals with UC relative to that observed in the healthy population [33]. In contrast, AVCP (300, 600, and 900 mg/kg/day) treatment considerably reduced the relative abundance of Bacteroides and increased the relative abundance of Firmicutes in colitis mice (Figure 8C,D), indicating a positive regulatory effect of AVCP on the composition of the gut microbiota. Noteworthy, low and medium doses of AVCP had a more significant modulating effect on Firmicutes and Bacteroides in colitis mice than high doses. At the genus level, there were 30 major genera in all the samples (Figure 8E).

**Figure 9:**
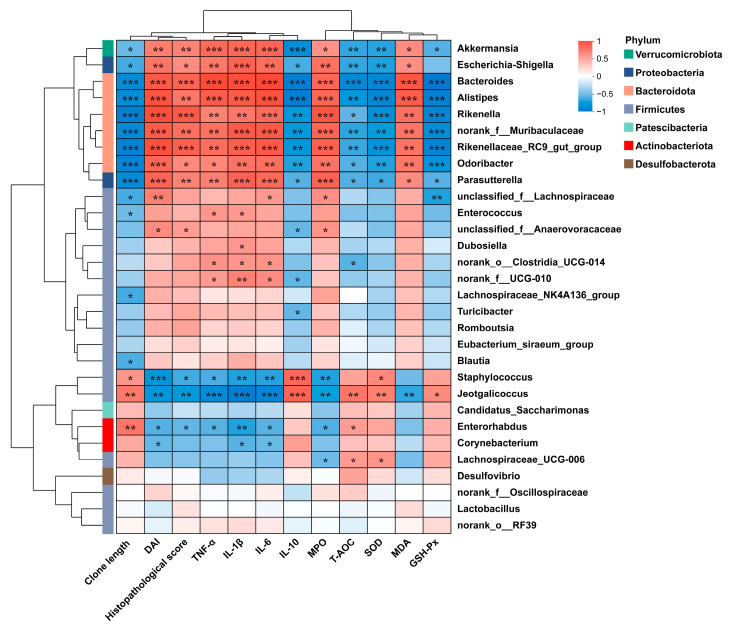
Correlation analysis between the top 30 abundances genera in all samples and inflammation-associated parameters. The red and blue blocks represent the positive and negative correlations, respectively. * *p* < 0.05, ** *p* < 0.01, and *** *p* < 0.001.

**Figure 10:**
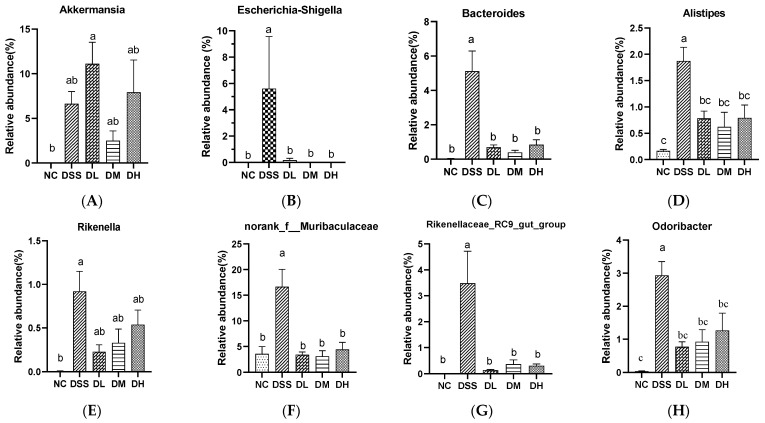

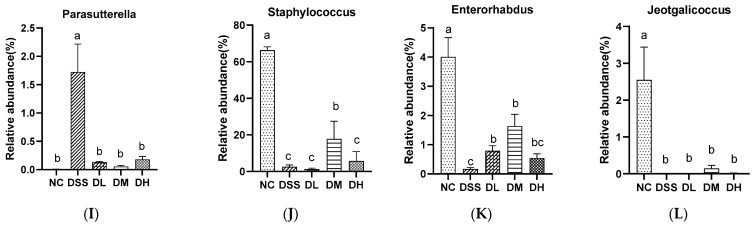
Relative abundance of dominant bacteria at the genus level. (**A**) *Akkermansia*; (**B**) *Escherichia-Shigella*; (**C**) *Bacteroides*; (**D**) *Alistipes*; (**E**) *Rikenella*; (**F**) *norank_f_Muribaculaceae*; (**G**) *Rikenellaceae_RC9_gut_group*; (**H**) *Odoribacter*; (**I**) *Parasutterella*; (**J**) *Staphylococcus*; (**K**) *Enterorhabdus*; (**L**) *Jeotgalicoccus*. Different lowercase letters indicate significant differences between groups (*p* < 0.05).

The heatmap of the correlation between the 30 major genera and the intestinal inflammatory-related parameters is shown in Figure 9. *Akkermansia*, *Escherichia-Shigella*, *Bacteroides*, *Alistipes*, *Rikenella*, *norank_f_Muribaculaceae*, *Rikenellaceae_RC9_gut_group*, *Odoribacter*, and *Parasutterella* abundances were significantly positively correlated with the DAI; histopathological score; and TNF-α, IL-1β, IL-6, MPO, and MDA levels, whereas negatively related to colon length, IL-10 level, T-AOC, and SOD and GSH-Px activity. The relative abundances of *Staphylococcus*, *Jeotgalicoccus*, and *Enterorhabdus* were significantly negatively correlated with the DAI; histopathological score; and TNF-α, IL-1β, IL-6, and MPO levels while positively related to colon length, IL-10 levels, T-AOC, and SOD and GSH-Px activity. The supplementation of AVCP significantly reduced the relative abundance of *Escherichia-Shigella*, *Bacteroides*, *Alistipes*, *Rikenella*, *norank_f_Muribaculaceae*, *Rikenellaceae_RC9_gut_group*, *Odoribacter*, and *Parasutterella* and decreased the *Staphylococcus*, *Jeotgalicoccus*, and *Enterorhabdus* abundance in colitis mice but had no significant effect on the relative abundance of *Akkermansia* (Figure 10). Moreover, AVCP has the most significant regulation of *Escherichia-Shigella*, *Bacteroides*, *norank_f_Muribaculaceae*, *Rikenellaceae_RC9_gut_group*, and *Parasutterella* in colitis mice and even brought them to normal levels (Figure 10B,C,F,G,I). These results indicate that AVCP significantly restores DSS-induced gut microbiota dysbiosis, particularly restoring the changes in *Escherichia-Shigella*, *Bacteroides*, *norank_f_Muribaculaceae*, *Rikenellaceae_RC9_gut_group*, and *Parasutterella* abundance.

## 4. Discussion

Research into the effects of collagen protein dietary supplementation on gut health has generated considerable interest. Extensive studies on the UC therapeutic effects of collagen peptides from fish or fish by-products have been conducted [22,23,34], whereas studies on collagen peptides from abalone viscera sources, and their effects on intestinal flora are rather scarce. In this study, collagen peptides from abalone viscera effectively alleviated body weight loss and fecal bleeding induced by the administration of a 2.5% DSS solution and decreased the DAI score that directly indicates the severity of UC. AVCP can also considerably ameliorate colon shortening, splenomegaly, thymic atrophy, and pathological damage colon. Moreover, the low-dose collagen peptides group showed less inflammatory cell infiltration compared to the high-dose group, contrary to previous studies that have suggested that higher doses are usually more effective [22,23]. However, Chen et al. demonstrated that a high-protein diet exacerbated colitis caused by DSS independent of protein composition [35]. This may be due to the fact that a high-protein diet increases mucinolytic bacteria and results in a thinner mucus layer [35].

Although the precise etiology of UC remains to be fully elucidated, it is strongly associated with the imbalance between pro-inflammatory and anti-inflammatory cytokines, excessive damage to the antioxidant defenses, and dysbiosis of the gut microbiota. Pro-inflammatory cytokines, including IL-1β, IL-6, and TNF-α, are expressed at relatively higher levels in the intestinal tissue and serum of patients with UC [28]. These cytokines were markedly decreased during UC recovery. In the current study, AVCP administration significantly reduced the elevated serum levels of IL-1β, IL-6, TNF-α, and IL-17A induced by DSS in mice, which suggests that AVCP can reduce inflammation in colitis mice. IL-10 is an anti-inflammatory cytokine that has been proven to exert a protective effect against UC [36]. The serum level of IL-10 was significantly increased following the administration of AVCP in colitis mice. Changes in the serum cytokines suggest that AVCP can partially restore the imbalance between pro-inflammatory and anti-inflammatory cytokines. Simultaneously, the alterations in MPO activity within the serum of mice were evaluated. MPO is a biomarker of neutrophil aggregation and correlates with the activity of inflammation [37]. Expectedly, AVCP markedly reduced the serum MPO levels in colitis mice, which further supports the fact that AVCP exerts a potential therapeutic effect against UC.

Pro-inflammatory cytokines have long been implicated in the production of reactive oxygen species (ROS) by immune cells and lead to oxidative stress [38]. Excessive release of ROS and sustained accumulation of oxidative stress weaken the immune system and contribute to immune-related diseases such as UC [39]. Thus, the T-AOC of serum is always reduced in patients with UC [40]. MDA is a marker of oxidative stress, mainly produced by ROS-induced lipid peroxidation, and readily cross-links with proteins, lipids, and nucleic acids to exacerbate cell membrane damage [41]. To prevent oxidative damage caused by oxidative stress, the most powerful system of an antioxidant defense system consisting of a combination of antioxidant enzymes such as SOD, GSH-Px, and CAT was developed [42]. Moreover, UC treatment can be targeted through the use of antioxidants, including glutathione, vitamin C, and vitamin E [31]. In this study, AVCP considerably decreased the MDA level and elevated the T-AOC, SOD, and GSH-Px levels in colitis mice serum. This suggests that AVCP may act as an antioxidant for improving the oxidative stress status in alleviating DSS-induced UC in mice.

Imbalances in the microbiota can lead to gut dysfunction and subsequent potential for UC [43]. In UC patients, the diversity and richness of gut microbiota are generally reduced with a rise in potentially harmful microorganisms and a decrease in beneficial bacteria compared to healthy people [44]. Conversely, our 16S rRNA gene sequencing results showed a significantly elevated α-diversity in the intestines of colitis mice. Current studies also showed that the gut flora in colitis mice was significantly richer and more diverse [45,46], which may be caused by the increased potentially harmful bacteria, including *Escherichia-Shigella*, *Parabacteroides*, and *Enterococcus*. At the phylum level, the gut of colitis mice contained fewer Firmicutes bacteria and more Bacteroidetes bacteria, and the ratio of F/B was markedly reduced. Similar studies have also shown that UC is associated with reduced Firmicutes and increased Bacteroidetes in the gut, and patients with more severe disease usually present a lower F/B ratio [47]. The increased Bacteroidetes abundance and decreased Firmicutes abundance, as well as F/B ratio bacteria, were significantly inhibited in the AVCP-treated groups.

At the genus level, the increased relative abundance of *Akkermansia*, *Escherichia-Shigella*, *Bacteroides*, *Alistipes*, *Rikenella*, *norank_f_Muribaculaceae*, *Rikenellaceae_RC9_gut_group*, *Odoribacter*, and *Parasutterella* was associated with the rise in the DAI; histopathological score; and TNF-α, IL-1β, IL-6, MPO, and MDA levels, as well as the decrease in colon length shortening, IL-10 level, T-AOC, and SOD and GSH-Px activity. In contrast, the relative abundance of *Staphylococcus*, *Jeotgalicoccus*, and *Enterorhabdus* showed an opposite trend in correlation with these indicators of inflammation. Among them, AVCP modulated *Escherichia-Shigella*, *Bacteroides*, *norank_f_Muribaculaceae*, *Rikenellaceae_RC9_gut_group*, and *Parasutterella* most significantly and even brought them to the normal level. The relative abundance of *Escherichia-Shigella* was significantly higher in the active UC group as compared to the non-IBD group but decreased in remitted UC [48]. Moreover, *Escherichia-Shigella* has been reported to promote the production of pro-inflammatory cytokines, including IFN-γ and IL-17 [49]. *Bacteroides,* particularly *B. vulgatus,* were observed abundantly in the colonic mucosal microbiota of patients with clinically active UC, and the administration of *B. vulgatus* in mice increased the intestinal permeability and induced colitis [50,51]. An increase in *Muribaculaceae* abundance was reported to be beneficial for gut homeostasis [52]. For UC patients, *Rikenellaceae* was significantly decreased in abundance [53]. However, in the DSS-induced mouse model of colitis, colitis was associated with a higher abundance of *Muribaculaceae* and *Rikenellaceae* [54]. *Parasutterella* was a crucial potentially pathogenic bacterium that was strongly associated with the progress of UC [55,56]. *Parasutterella* was more increased in aged UC patients with severe colitis than those of younger UC patients [57]. In the present study, we found that AVCP significantly inhibited the proliferation of *Escherichia-Shigella*, *Bacteroides*, *norank_f_Muribaculaceae*, *Rikenellaceae_RC9_gut_group*, and *Parasutterella* in colitis mice. These findings on the gut microbiota suggest that AVCP can modulate the balance of the gut microbiota and suppress potentially pathogenic bacteria, thereby becoming a potential therapy for ulcerative colitis.

## 5. Conclusions

In summary, our study suggested that collagen peptides derived from abalone viscera can effectively alleviate DSS-induced mice acute colitis symptoms, including weight loss, colon length shortening, and bloody diarrhea. The ameliorative effects of AVCP may be the result of the down-regulation of pro-inflammatory cytokines, enhancement of intestinal antioxidant capacity, and restoration of gut microbiota disorders. However, further studies are necessary for the elucidation of the ameliorative mechanisms of AVCP in UC.

## Figures and Tables

**Table 1:** Molecular weight distribution and hydroxyproline content of AVCP.

Molecular Weight Distribution		Hydroxyproline Content
<1000 Da	19.3%	6.56 ± 0.23%
1000–2000 Da	21.0%	
2000–3000 Da	25.6%	
3000–5000 Da	23.1%	
5000–10,000 Da	8.1%	
>10,000 Da	2.9%	

## Data Availability

The raw sequence data of 16S rRNA sequencing of the gut microbiota has been deposited in the NCBI Short Read Archive database under the BioProject accession number PRJNA1236735 (data will be released on 28 February 2029).

## Supplementary Materials

The following supporting information can be downloaded at https://www.mdpi.com/article/10.3390/nu17111926/s1: Figure S1: H&E staining of representative histology sections of (A) heart, (B) liver, (C) spleen, (D) lung, (E) kidney. (40×, 200×, scale bar 100 μm).; Figure S2: The serum levels of (A) IL-1β, (B) IL-6, (C) TNF-α, (D) MDA, and (E) SOD in mice.; Table S1: Average weight change of mice in each week; Table S2: Effect of AVCP on organ index in normal mice.

## Abbreviations

The following abbreviations are used in this manuscript:

AVCP	abalone viscera collagen peptide
UC	ulcerative colitis
IBD	inflammatory bowel disease
DSS	dextran sulfate sodium
DAI	disease activity index
MPO	myeloperoxidase
TNF-α	tumor necrosis factor-α
IL	interleukins
MDA	malondialdehyde
T-AOC	total antioxidant capacity
SOD	superoxide dismutase
GSH-Px	glutathione peroxidase
PCoA	principal coordinate analysis
NMDS	non-metric multidimensional scaling
