# Supplementary material for: The Alleviating Effect of Abalone Viscera Collagen Peptide in DSS-Induced Colitis Mice: Effect on Inflammatory Cytokines, Oxidative Stress, and Gut Microbiota
Source: Nutrients. 2025 Jun 4;17(11):1926. doi: 10.3390/nu17111926 (PMC12158132; doi:10.3390/nu17111926)
Supplement: Supplementary file 1 [file nutrients-17-01926-s001.zip › nutrients-3565983-supplementary.pdf]

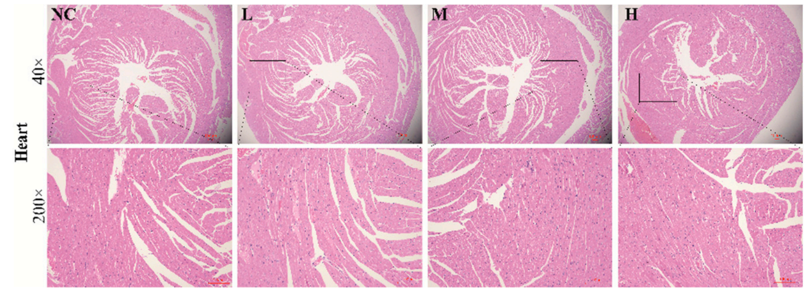

(A)

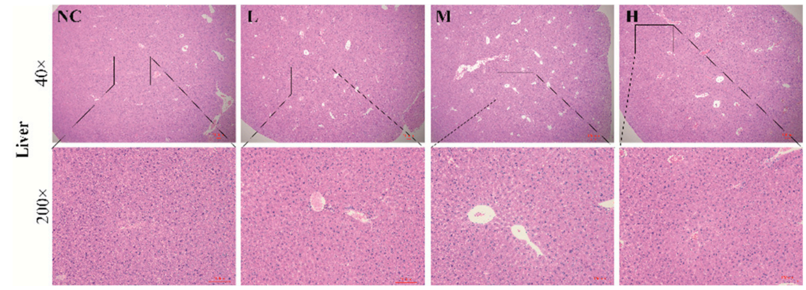

(B)

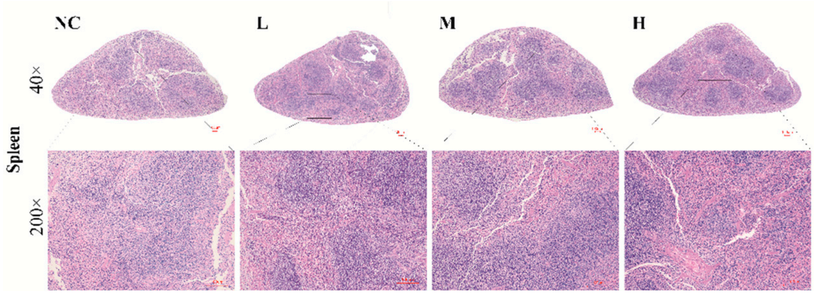

(C)

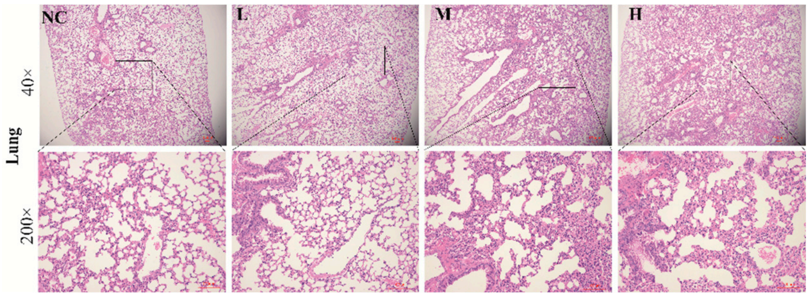

(D)

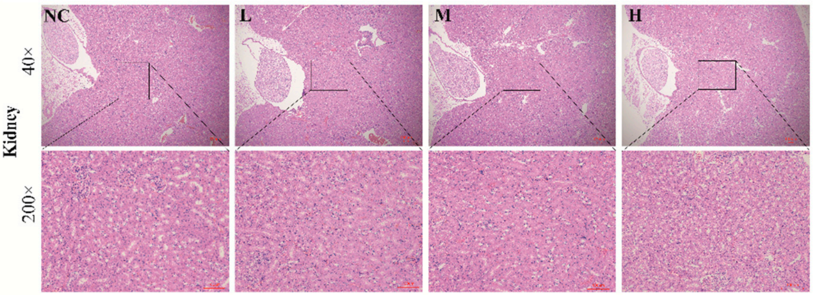

(E)

Supplementary Figure S1. H&E staining of representative histology sections of (A) heart, (B) liver, (C) spleen, (D)lung, (E) kidney.(40×, 200×, scale bar 100 μm).

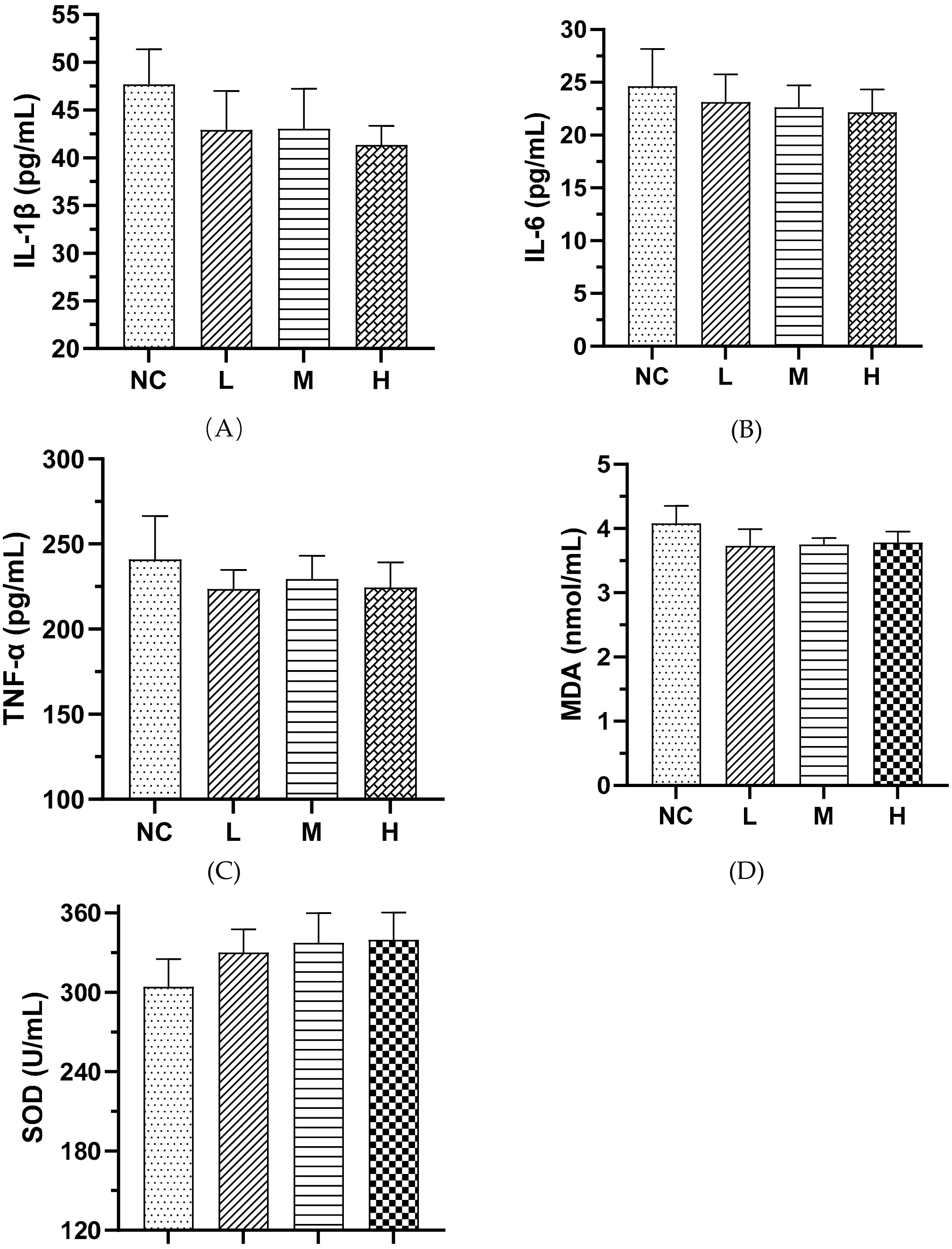

Supplementary Figure S2. The serum levels of (A) IL-1β, (B) IL-6, (C) TNF-α, (D) MDA, and (E) SOD in mice.

Supplementary Table S1. Average weight change of mice in each week

Groups	Weight (g)			
	First week	Second week	Third week	Fourth week
NC	22.76±1.55	23.61±1.99	24.34±1.92	24.77±2.10
L	21.94±1.09	23.28±1.2	24.03±1.64	24.68±1.60
M	21.96±0.74	23.12±1.39	24.41±1.70	25.13±1.69
H	21.93±0.74	23.33±1.13	24.19±1.141	24.92±1.06

NC: normal control; L, M, H: mice gavaged with 300mg/kg, 600 mg/kg, 900 mg/kg of AVCP, respectively.

Supplementary Table S2. Effect of AVCP on organ index in normal mice

Groups	Organ index (%)			Immune organ index (mg/g)	
	Heart	Liver	Kidney	Spleen	Thymus
NC	0.63±0.06	4.36±0.35	1.23±0.07	2.25±0.20	1.42±0.44
L	0.65±0.13	4.51±0.28	1.31±0.06	2.52±0.16	1.61±0.48
M	0.61±0.10	4.55±0.27	1.25±0.08	2.44±0.14	1.54±0.31
H	0.60±0.11	4.24±0.28	1.29±0.14	2.41±0.39	1.49±0.44

NC: normal control; L, M, H: mice gavaged with 300mg/kg, 600 mg/kg, 900 mg/kg of AVCP, respectively.
